# *Clostridium difficile* Is an Autotrophic Bacterial Pathogen

**DOI:** 10.1371/journal.pone.0062157

**Published:** 2013-04-23

**Authors:** Michael Köpke, Melanie Straub, Peter Dürre

**Affiliations:** 1 Institut für Mikrobiologie und Biotechnologie, Universität Ulm, Ulm, Germany; 2 LanzaTech, Auckland, New Zealand; Charité, Campus Benjamin Franklin, Germany

## Abstract

During the last decade, *Clostridium difficile* infection showed a dramatic increase in incidence and virulence in the Northern hemisphere. This incessantly challenging disease is the leading cause of antibiotic-associated and nosocomial infectious diarrhea and became life-threatening especially among elderly people. It is generally assumed that all human bacterial pathogens are heterotrophic organisms, being either saccharolytic or proteolytic. So far, this has not been questioned as colonization of the human gut gives access to an environment, rich in organic nutrients. Here, we present data that *C. difficile* (both clinical and rumen isolates) is also able to grow on CO_2_+H_2_ as sole carbon and energy source, thus representing the first identified autotrophic bacterial pathogen. Comparison of several different strains revealed high conservation of genes for autotrophic growth and showed that the ability to use gas mixtures for growth decreases or is lost upon prolonged culturing under heterotrophic conditions. The metabolic flexibility of *C. difficile* (heterotrophic growth on various substrates as well as autotrophy) could allow the organism in the gut to avoid competition by niche differentiation and contribute to its survival when stressed or in unfavorable conditions that cause death to other bacteria. This may be an important trait for the pathogenicity of *C. difficile*.

## Introduction

*Clostridium difficile* represents a considerable threat to the European and North American healthcare systems. Infection rates show a constant rise, and hypervirulent strains led to numerous nosocomial outbreaks [1]. The significance of the disease is also stressed by the enormous rise of respective scientific publications during the last 10 to 15 years. *C. difficile* is meanwhile the major cause of diarrhea and colitis in developed countries [2], with at least 5000 deaths per year in the United States [3]. Major virulence factors for these diseases are glycosylating toxins A and B, also referred to as large clostridial toxins. Both exert cytotoxic activity and corresponding virulence [4]. Treatment of the disease is hampered by the fact that *C. difficile* is able to generate endospores, highly resistant bacterial survival forms, which thus can persist in the gut after antibiotic treatment, germinate again into viable cells, and lead to recurrence of the disease.

**Figure 1:**
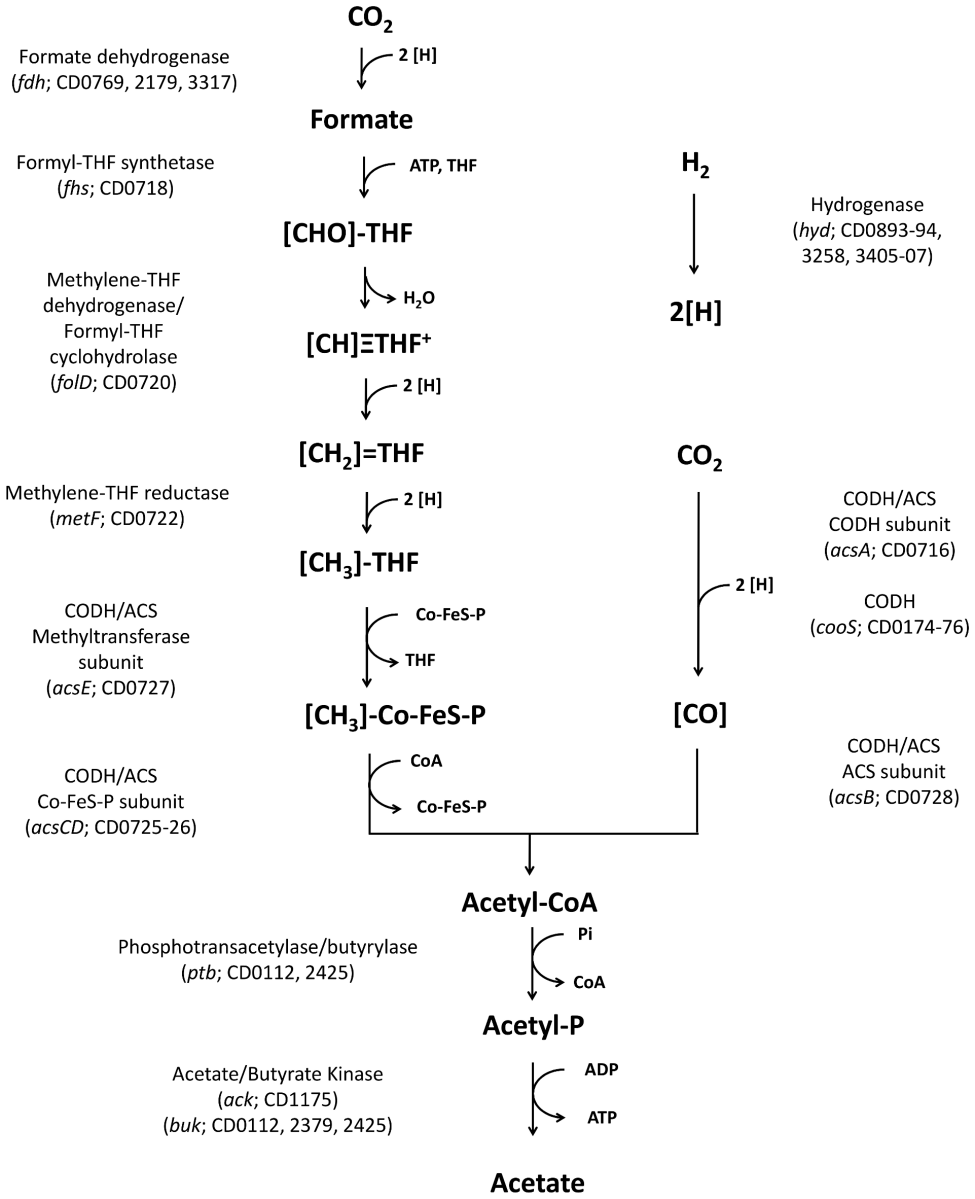
Wood-Ljungdahl pathway and involved genes of *C. difficile* 630. ACS, acetyl-CoA synthase; CODH, carbon monoxide dehydrogenase; CoFeS, corrinoid-iron-sulfur protein; THF, tetrahydrofolate.

As of yet, all human bacterial pathogens (thus including *C. difficile*) are considered to be heterotrophic organisms [5], feeding either on starch and sugars (saccharolytic) or proteins and peptides (proteolytic). These substrates are fermented in the gut mostly to organic acids and the gases carbon dioxide and hydrogen. Under anaerobic conditions, two pathways are well known, which can make use of such gas mixtures. Methanogens, microorganisms belonging to the Archaea, produce methane. In methanogens, it was estimated that a total of more than 200 genes were required for autotrophic growth on CO_2_ and H_2_ including biosynthesis, co-factor and energy conservation [6]. Acetogens, representing eubacteria, employ the Wood-Ljungdahl pathway (Fig. 1) to convert CO_2_+H_2_ into acetate (and sometimes other compounds such as ethanol, 2,3-butandiol, butanol, and/or butyrate as well) [7]–[11]. The reductive acetyl-CoA or Wood-Ljungdahl pathway is the only linear CO_2_ fixation pathway known and speculated to be one of the first biochemical pathways existing on earth [12]. The model organism for elucidation of the respective enzymatic steps was *Moorella thermoacetica* (formerly *Clostridium thermoaceticum*) [13], which was originally isolated under heterotrotrophic conditions. In this report we show that Wood-Ljungdahl pathway genes are present and conserved in all sequenced *C. difficile* strains to date and that clinical isolate and model strain *C. difficile* 630 as well as related isolates are able to grow autotrophically. Thus, *C. difficile* represents the first identified bacterial pathogen with this metabolic trait, giving the organism great metabolic flexibility in the gut environment, not only feeding on sugars and proteins but potentially also on CO_2_ and H_2_ produced by other organisms.

## Materials and Methods

### Bacterial Strains and Growth Conditions

*C. difficile* 630 (ATCC BAA-1382™) was obtained from the American Type Culture Collection (ATCC), Manassas, VA, USA and *C. difficile* DSM 1296, DSM 12056, and DSM 12057 from Deutsche Sammlung von Mikroorganismen und Zellkulturen GmbH (DSMZ), Braunschweig, Germany.

All organisms were cultivated anaerobically at 37°C and growth was monitored by measuring the optical density at 600 nm (OD_600 nm_).

Reviving of stock cultures was performed in reinforced clostridial medium RCM (BD, Franklin Lakes, NJ, USA). For solid media, 1.2% (w/v) Bacto agar was used (BD, Franklin Lakes, NJ, USA).

For growth experiments, 50 mL medium were used, either PETC medium (omitting yeast extract) [14] or 50 mL AC-11 medium (omitting yeast extract) [15]. Medium was prepared using strictly anaerobic methods. All chemicals were purchased by Sigma-Aldrich, Schnelldorf, Germany or Merck KGaA, Darmstadt, Germany.

All growth experiments were carried out in triplicates with three biological replicates using 1-L bottles with 0.8 bar (gauge) of either a mix of CO_2_+H_2_ (20∶80) or CO as headspace, or 20 mM of a mixture of glucose and fructose (50∶50) (under an N_2_ atmosphere) as substrate. Medium (without a carbon source) and 0.8 bar (gauge) N_2_ as headspace (rather than CO_2_+H_2_, or CO) has been used as control.

The inoculum was prepared as follows: For strains *C. difficile* 630 and *C. difficile* type strain DSM 1296, a 5-mL overnight culture grown in RCM medium was washed twice with anaerobic PETC medium and then used for inoculation. Acetogenic isolates DSM 12056 and DSM 12057 were grown in 50 mL AC-11 medium (including 0.5 g/L yeast extract and a 50∶50 mixture of 20 mM glucose and fructose), until exponential growth phase (after 2 days) as described earlier [15], then washed once with AC-11 medium without yeast extract and used to inoculate at an OD_600 nm_ of 0.1. *C. difficile* 630 and *C. difficile* were grown in PETC medium, and acetogenic isolates DSM 12056 and DSM 12057 in AC-11 medium.

Growth was followed by biomass measurements throughout the growth, drop of pressure in the headspace (measured with a syringe), and level of metabolites at end of growth.

### Detection of Metabolites

The produced metabolites were quantified by a gas chromatograph equipped with a flame ionization detector (Clarus 600, Perkin Elmer, Waltham, MA, USA). 2-ml samples were taken from the bacterial culture, centrifuged (10000×g, 10 min), and the supernatant was used for detection. The sample volume was 1 µl and isobutanol was used as an internal standard. Separation of the metabolites was carried out on a Chromosorb 101 packed glass column (80–100 mesh; 2 mm diameter; 2 m length). N_2_ was used as carrier gas (15 ml/min). The injection temperature was 195°C and the GC oven had a temperature profile of 130°C for 1 min, 130–200°C with 4°C increase per minute, and finally 200°C for 3 min. The detector was maintained at 300°C. Ethanol, acetate, butyrate, isovalerate, and isocaproate were detected.

### Bioinformatics

Wood-Ljungdahl pathway sequences were identified using Basic Local Alignment Search Tool (BLAST) [16], Artemis Comparison Tool (ACT) [17] and Geneious (Biomatters Ltd., New Zealand). Genes for *C. difficile* genome sequences without annotation (BI9, CF5, M68, M120, 2007885) were predicted using Glimmer [18].

## Results and Discussion

### DNA Sequence Comparisons

**Figure 2:**
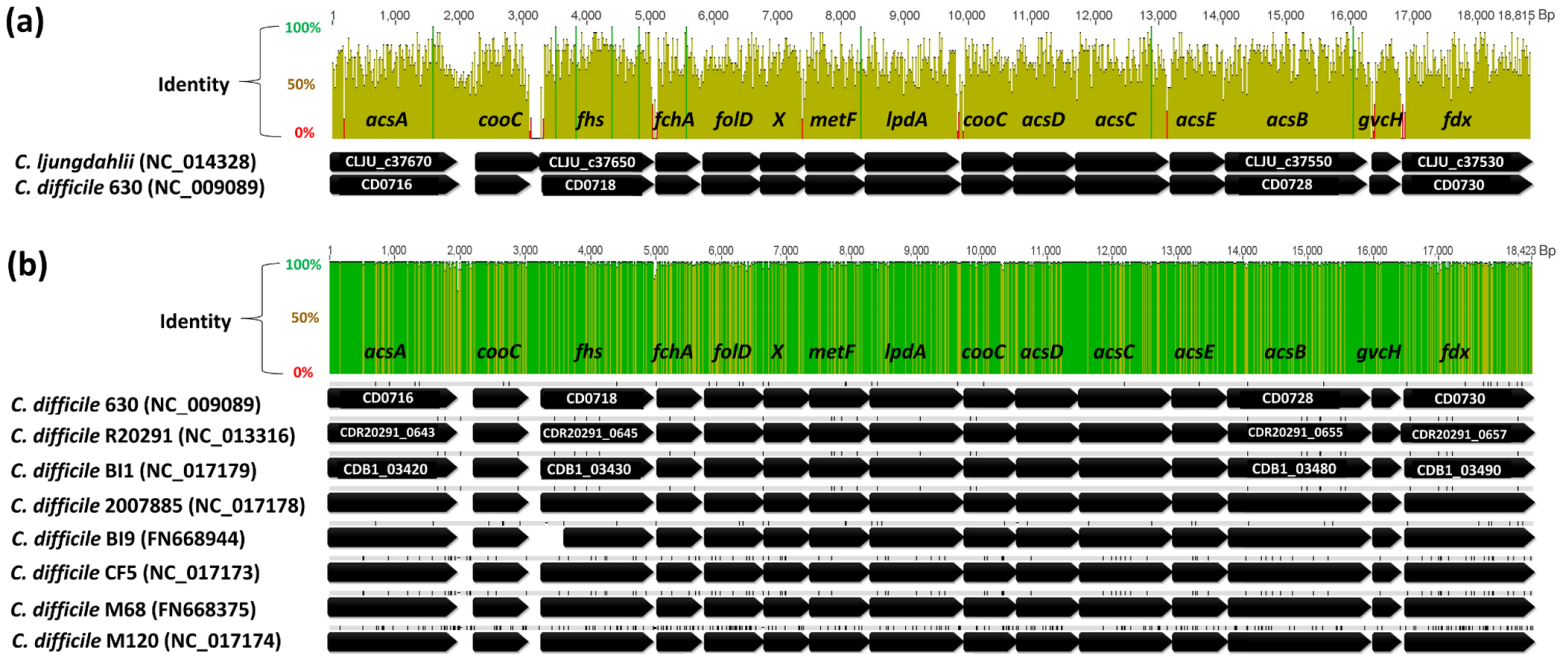
Genetic arrangement of Wood-Ljungdahl-pathway genes in **C. ljungdahlii** and *C. difficile*: (a) Alignment of *C. ljungdahlii* and *C. difficile* 630 and (b) alignment of sequenced *C. difficile* strains against each other. Sequence identity is represented by colored graphs above the alignments, variations and gaps to the consensus sequence are highlighted in black above the respective sequences. Locus numbers are given for annotated sequences. *acs*, genes for the CODH/ACS complex; *acsA*, CODH subunit gene; *acsB*, ACS subunit gene; *acsC*, CoFeS large subunit gene; *acsD*, CoFeS small subunit gene; *acsE*, methyltransferase subunit gene; *cooC*, gene for CODH accessory protein; *fchA*, formimimo-THF cyclodeaminase gene; *fdx*, ferredoxin gene; *fhs*, formyl-THF synthetase gene; *folD*, bifunctional methylene-THF dehydrogenase/formyl-THF cyclohydrolase gene; *gcvH*, gene for glycine cleavage system H protein; *lpdA*, gene for dihydrolipoamide dehydrogenase; *metF*, methylene-THF reductase gene; *X*, hypothetical gene.

During annotation of the genome of *Clostridium ljungdahlii* [14], an acetogenic bacterium able to use gases CO and/or CO_2_+H_2_ as substrate [19], we realized that the respective Wood-Ljungdahl pathway genes enabling autotrophic growth (Fig. 1) are also present in the reported genome sequence of clinical isolate *C. difficile* 630 [20], [21], arranged in exactly the same order (CD0716-30 of *C. difficile* strain 630) (Fig. 2). Meanwhile full genome sequences of eight other *C. difficile* strains have become available (human strains BI1 [22], BI9 [22], CD196 [23], CF5 [22], M68 [22], M120 [22], R20291 [23], and bovine strain 2007885), as well as draft genome sequences of 19 other clinical isolates (strains 6534, 6407, 6466, 6503, 002-P50-2011, 050-P50-2011, 70-100-2010, ATCC 43255, CD37, CIP 107932, NAP07, NAP08, QCD-23m63, QCD-32g58, QCD-37×79, QCD-63q42, QCD-66c26, QCD-76w55, QCD-97b34). The region of the Wood-Ljungdahl pathway genes has been found to be present and highly conserved in all sequenced strains (Table 1), despite the diverse and dynamic nature of the *C. difficile* genome [22] (Fig. 2). The genes for the key enzyme of acetogens, the bifunctional carbon monoxide dehydrogenase/acetyl-CoA synthase complex (CODH/ACS), are part of this cluster. In addition, a gene for another monofunctional carbon monoxide dehydrogenase (CODH) gene has been found in the genomes of all sequenced *C. difficile* strains. As in *C. ljungdahlii*, this gene is in an operon with genes for an electron transfer protein and an oxidoreductase, which may form a complex (CD0174-76) (Table 1). Functional hydrogenase and formate dehydrogenase are also required for growth on CO_2_+H_2_. All analyzed *C. difficile* strains contain at least two non-seleno formate dehydrogenases (CD0769, 2179), but interestingly only *C. difficile* strain 630 and CD196 also a predicted seleno formate dehydrogenase with a SECIS (selenocysteine integration sequence) element (CD3317) [24]. Four Fe-only hydrogenases (CD0893, 0894, 3258, 3405-07) are conserved in all strains, from which one (CD3405-07) resembles an electron-bifurcating hydrogenase type as discovered in *Thermotoga maritima* [25]. Remarkably, two of the hydrogenases genes (CD0893, 0894) are directly adjacent and given the high sequence identity likely a result of gene duplication, nevertheless the same arrangement is fully conserved in all analyzed *C. difficile* strains. In addition, genes for an Rnf complex (CD1137-42) that is speculated to be the coupling site for energy conservation in acetogens without cytochromes during autotrophic growth are also present [14], [26], [27]. All genes are highly conserved between the sequenced *C. difficile* strains and are located at the similar loci in the genome as shown in Table 1.

**Table 1:** Overview and organization of Wood-Ljungdahl pathway, CODH, formate dehydrogenase, hydrogenase, and Rnf complex genes in sequenced and annotated *C. difficile* strains (ORF numbers according to their original annotation and position in the genome is given) and homologues in *C. ljungdahlii* (ORF number and identity on protein level against *C. difficile* strain 630 is given).

	*C. difficile* 630	*C. difficile* CD196	*C. difficile* R20291	*C. difficile* BI1	*C. ljungdahlii*
	(CD)	(CD196_)	(CDR20291_)	(CDBI1)	(CLJU_c)
**CODH cluster**	**0174–76**	**0188–90**	**0175–77**	**00950–60**	**09090–9110**
	(230,672…234,240)	(228,785…232,353)	(226,209…229,777)	(238,442…242,010)	(52–69% AA identity)
**Wood-Ljungdahl cluster**	**0716–30**	**0661–76**	**0643–57**	**03420–90**	**37670–37530**
**(including CODH/ACS)**	(876,288…894,710)	(802,336…820,758)	(799,876…818,298)	(811,890…830,312)	(59–75% AA identity)
**Formate dehydrogenase 1**	**0769**	**0717**	**0698**	**3690**	**15540**
**(non-seleno)**	(940,287…942,431)	(868,564…870,876)	(866,179…868,491)	(878,342…880,654)	(45% AA identity)
**Hydrogenase 1+2**	**0893–94**	**0843–44**	**0823–24**	**04320–325**	**37220**
**(gene duplication)**	(1,074,603…1,077,908)	(1,004,321…1,007,604)	(1,001,937…1,005,220)	(1,014,138…1,017,382)	(54% AA identity)
**Rnf complex**	**1137–42**	**0995–1000**	**0973–78**	**05090–115**	**11360–410**
**(RnfCDGEAB)**	(1,336,456…1,341,545)	(1,194,616…1,199,705)	(1,192,232…1,197,321)	(1,204,393…1,209,482)	(32–50% AA identity)
**Formate dehydrogenase 2**	**2179**	**2042**	**2085**	**10575**	**8930**
**(non-seleno)**	(2,521,529…2,519,352)	(2,361,784…2,359,607)	(2,442,337…2,440,160)	(2,369,741…2,367,618)	(25% AA identity)
**Hydrogenase 3**	**3258**	**3070**	**3116**	**15955**	**20290**
	(3,816,870…3,815,434)	(3,642,515…3,641,061)	(3,723,290…3,721,836)	(3,650,517…3,649,081)	(39% AA identity)
**Formate dehydrogenase 3**	**3317**	**3133**	**not present**	**not present**	**CLJU_c06990**
**(seleno)**	(3,816,870…3,815,434)	(3,711,229…3,713,373)			(73% AA identity)
**Hydrogenase 4**	**3405–07**	**3181–83**	**3227–29**	**16535–545**	**14700–720**
**(** ***T. maritima*** ** type)**	(3,983,987…3,988,191)	(3,768,584…3,772,788)	(3,849,368…3,853,572)	(3,776,602…3,780,806)	(57–59% AA identity)

### Growth Under Autotrophic Conditions

**Figure 3:**
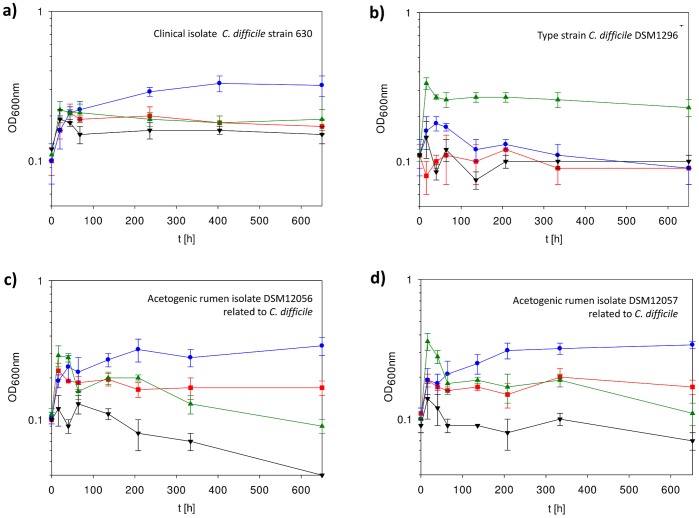
Growth of various *C. difficile* strains in chemically defined acetogenic media under autotrophic and heterotrophic conditions. Red squares, CO as sole carbon and energy source; Blue circles, CO_2_+H_2_ as sole carbon and energy source; Green triangles up, glucose and fructose as substrates; Black triangles down, control (N_2_). (a) *C. difficile* clinical isolate 630 (ATCC BAA-1382™), (b) *C. difficile* type strain DSM 1296, (c) acetogenic isolate DSM 12056, (d) acetogenic isolate DSM 12057. Error bars represent standard deviation.

The presence of genes required for autotrophy came as a surprise, as *C. difficile* was isolated and always cultivated on rich media containing organic substrates. Presence and expression of Wood-Ljungdahl pathway genes serves as classification as an acetogen [28]. We carried out growth experiments with the sequenced strain, clinical isolate *C. difficile* 630 (ATCC BAA-1382™) [20], [21], [29] in typical chemically defined media used for acetogens containing no other carbon sources to find out if these genes are indeed functional. The organism grew poorly with gas mixtures as only carbon and energy source compared to growth in rich complex media. Nevertheless, we could observe slight growth on CO_2_+H_2_ (2 doublings), while almost no growth was observed, when CO_2_+H_2_ was replaced by either CO or N_2_ or with sugars (glucose+fructose) as substrate (1 doubling or less) (Fig. 3a). Furthermore, only cultures grown on CO_2_+H_2_ continued to grow (up to an OD_600 nm_ of 0.2) when sub-cultured into fresh identical media (a pressure drop was only observed in cultures grown with CO_2_+H_2_, not in cultures gassed with N_2_) and also produced significant amounts of acetate in contrast to the other cultures (0.67 g/L acetate formed with CO_2_+H_2_ over 0.22 g/L with fructose; Table 2). Acetate production is a striking feature of acetogens, reflected in their name, and all acetogenic species described to date have been shown to produce acetate. An operon with acetate biosynthesis genes phosphotransacetylase (*pta*) and acetate kinase (*ack*) is found in *C. ljungdahlii* [14] and all other acetogens sequenced to date such as *Moorella thermoacetica* [30], *Acetobacterium woodii* [27], *Eubacterium limosum* [31], or *C. carboxidivorans* [32]. Interestingly, only an orphan acetate kinase gene is found in the *C. difficile* 630 genome (CD1175), but no gene for a phosphotransacetylase. There are however two phosphotransbutyrylase-butyrate kinase (*ptb-buk*) cluster (CD0112-13; CD2425-26), as well as an additional phosphotransbutyrylase (CD0715) and butyrate kinase (CD2379), which may be unspecific enough to accept both acetyl-CoA and butyryl-CoA (respectively the corresponding phosphates). The same situation is found in other *C. difficile* strains. The lack of a specialized phosphotransacetylase enzyme may explain the poor growth obtained.

**Table 2:** Metabolites detected at end of growth in cultures of *C. difficile* strain 630, type strain DSM 1296, and acetogenic isolates DSM 12056 and DSM 12057 on different substrates (ND, not detected; error represents standard deviation).

Strain	Substrate	Metabolites produced at end of growth [g/L]		
		Acetate	Ethanol	Butyrate
*C. difficile* 630	CO	0.12±0.04	0.09±0.04	ND
	CO_2_+H_2_	0.67±0.07	0.03±0.01	ND
	Glucose+Fructose	0.22±0.01	0.10±0.03	0.12±0.01
	Control (N_2_)	0.05±0.03	0.03±0.01	ND
*C. difficile* DSM 1296	CO	0.09±0.02	0.04±0.01	ND
	CO_2_+H_2_	0.08±0.02	0.01±0.01	ND
	Glucose+Fructose	0.56±0.09	0.08±0.03	0.79±0.05
	Control (N_2_)	0.09±0.03	ND	ND
Acetogenic isolate DSM 12056	CO	0.11±0.04	0.12±0.03	0.02±0.01
	CO_2_+H_2_	0.79±0.13	0.02±0.01	ND
	Glucose+Fructose	0.87±0.09	0.23±0.06	0.56±0.12
	Control (N_2_)	0.09±0.03	0.03±0.01	ND
Acetogenic isolate DSM 12057	CO	0.06±0.03	0.07±0.02	ND
	CO_2_+H_2_	0.83±0.09	ND	ND
	Glucose+Fructose	0.90±0.10	0.21±0.06	0.49±0.12
	Control (N_2_)	0.07±0.02	0.01±0.01	ND

Next, we examined the autotrophic potential of further strains: the type strain *C. difficile* DSM 1296 [33] and two acetogenic isolates from rumen DSM 12056 (strain AA1) and DSM 12057 (strain A90) which are closely related to *C. difficile* according to 16S rRNA comparisons [15]. While the *C. difficile* type strain only grew on sugars, but not on CO_2_+H_2_ or CO (Fig. 3b), DSM 12056 and DSM 12057 were able to grow on both, sugar and CO_2_+H_2_ (Fig. 3c +3d). This was already described earlier [15], but only on AC11 media containing 0.5 g yeast extract/L. We omitted the yeast extract to ensure CO_2_+H_2_ is the sole source of carbon and energy, still achieving growth and comparable acetate production (Table 2) to what has been reported previously [15]. The strain was also able to grow in PETC media, although to a slightly reduced maximum biomass concentration (data not shown). In contrast, no growth occurred when CO_2_+H_2_ was replaced with N_2_ (Fig. 3b–3d). After transfer into fresh media with gases as carbon and energy source, CO_2_+H_2_ grown cultures of DSM 12056 and DSM 12057 showed stable growth and reached the same OD_600nm_ again over multiple generations. Depending on the substrate, the amount of products differs in those two strains. Cultures grown on CO_2_+H_2_ produced mainly acetate, while on fructose and glucose also butyrate was formed (Table 2).

Growth for both clinical *C. difficile* strain 630 and acetogenic rumen isolates DSM 12056 and DSM 12057 was only weak under autotrophic conditions, but might be improved by adaptation and by using a CO_2_:H_2_ mixture of 1∶2 (which is more favorable for acetogenic bacteria) and higher pressure (to have more gas dissolved in the liquid and achieve a better mass transfer). Given the fact that with sugars as substrate, *C. difficile* strain 630 hardly grows (less than 2 doublings) and the type strain DSM 1296 also only reached an OD_600nm_ of around 0.4, optimization of the media formulation may be required. A chemically defined media for *C. difficile* has been reported, but only for strains VPI 10463, KZ 1626, KZ 1630, KZ 1647 and KZ 1748, which require cysteine, isoleucine, leucine, proline, tryptophan, and valine for growth up to an OD_590nm_ of 0.8 [34], [35]. For clinical isolate *C. difficile* 630, no chemically defined media has been described and no auxotrophy is known from genome analysis [20], [21]. Supplementation of 1 g yeast extract/L did not enhance growth as determined in an initial experiment (data not shown). Thus, absence of amino acids is not a limiting factor for lack of autotrophy of *C. difficile* 630. Acetogenic rumen isolates DSM 12056 and DSM 12057 were growing similarly with CO_2_+H_2_ as sole carbon and energy source as described earlier in the presence of small amounts of yeast extract [15].

Further support for the *C. difficile* autotrophy comes from other acetogenic isolates, also from the ruminal reservoir of newborn lambs, that are also closely related to *C. difficile* according to 16S rRNA and DNA-DNA reassociation comparisons and were also able to grow on CO_2_+H_2_ [36].

From the tested strains, only the type strain DSM 1296 did not grow on CO_2_+H_2_. This strain was already isolated 1935 from infants [33]. It might well be that this strain lost the ability to grow autotrophically over the years by continued cultivation on complex media in various type collections. Knowledge of the genome sequence, which is not yet available, would allow one to determine whether the organism has lost (partially or in total) the Wood-Ljungdahl pathway genes or, in case they are still retained, whether they have been mutated or silenced. It should be noted that also the model organism *M. thermoacetica*, which had been used for elucidation of the Wood-Ljungdahl pathway, was only much later found to grow on CO_2_+H_2_ (only 10 out of 13 strains tested), at very low optical densities (up to app. 0.1 at 660 nm) [37]. The authors speculated on a loss of capacity for autotrophy.

### Concluding Remarks

In this report, we have shown that the sequenced strain of *C. difficile* (clinical isolate *C. difficile* 630 (ATCC BAA-1382™) [20], [21], [29] is a true acetogenic organism and able to grow autotrophically on CO_2_+H_2_ as sole carbon and energy source (no other carbon source present in defined media). This ability is based on the presence of Wood-Ljungdahl pathway genes in *C. difficile* 630, which have been found to be highly conserved in all other sequenced strains of *C. difficile* to date. Few transcriptomic studies of *C. difficile* strain 630 have been performed [38], [39], in which the Wood-Ljungdahl pathway genes have been shown to be expressed. In addition, the methyltransferase of strain 630 (encoded by CD0727) has been purified in recombinant *E. coli* and its activity been confirmed [40].

Acetogens are known for their energy efficiency, the use of a wide range of electron acceptors, and remarkable metabolic flexibility that enables them to survive even when stressed or in unfavorable conditions and to avoid competition via niche differentiation [41], [42]. The Wood-Ljungdahl pathway also provides an advantage under heterotrophic conditions, as CO_2_ produced during glycolysis can be fixed as additional carbon. It has been demonstrated that acetogens are abundant in the human gut, feeding from a variety of carbohydrates or the gases CO_2_ and H_2_ and accounting for approximately 35% of all acetate produced from carbohydrates [43] and 10^10^ kg of acetate per year from CO_2_ and H_2_ [44]. Taking the multidrug-resistance of several *C. difficile* strains into account, this metabolic flexibility renders *C. difficile* very persistent and difficult to eliminate, whereas it has been shown that *C. difficile* is outcompeted by other gut microorganisms when only specific carbohydrates are present [45]. Thus, the autotrophic capability may contributes to the severe pathogenicity of this organism and provides an explanation for the persistence of the organism compared to other spore formers. A proteome analysis of *C. difficile* strain VPI 10463 showed that during maximum toxin production, proteins of the the Wood-Ljungdahl pathway were found upregulated (from only 40 proteins found in total) [46]. The genes of the Wood-Ljungdahl pathway were also found upregulated during heat stress response [39]. Genetic tools for *C. difficile* strain 630 have recently been developed, including the generation of in-frame deletion mutants, and could help to identify the role of these genes and their involvement in pathogenicity of this organism [47].

While this is the first report to show growth of a bacterial pathogen under autotrophic conditions, it should be mentioned that some other species obviously also require CO_2_ for optimal growth. *Listeria monocytogenes* and *Yersinia pseudotuberculosis* consumed CO_2_ from a gas mixture (max. 3% CO_2_) and incorporated ^14^CO_2_ into cell material, but growth on gas mixtures was not documented [48]. Thus, the CO_2_ uptake might be due to activity of an anaplerotic enzyme, such as PEP-carboxylase. The third known example of CO_2_ consumption by a pathogen refers to *Mycobacterium leprae*. It has been suggested that the organism might be closer related to the genus *Nocardia* than to *Mycobacterium* and that CO_2_ is used [49], [50], but again no growth curves have been documented and the media obviously contained other carbon sources in addition to carbon dioxide. Thus, *C. difficile* remains to be the first example of a true autotrophic bacterial pathogen, able to grow with gases as CO_2_ and H_2_ as sole energy and carbon source.
